# Uses of the Pancreatic Juice in Digestion

**Published:** 1849-07

**Authors:** 


*Uses of the Pancreatic Juice in Digestion.*— Dr. Bernard lately read before the Academy of Sciences, a memoir on the uses of the pancreatic juice in digestion. The object of the author is to demonstrate experimentally that the pancreatic juice is destined, to the exclusion of all the other intestinal liquids, to modify in a special manner, or in other words, to digest, the neutral fats contained in the aliments, and thus allow their ulterior absorption by the chyliferous vessels. It results from experiments performed by Dr. Bernard to ascertain the physiological properties of the pancreatic juice within and without the living body:

1st. That the pancreatic juice possesses the property of emulsionizing instantly and completely the neutral fats, and afterwards of acidifying them and separating them into fatty acid and glycerine. The pancreatic juice alone of all the liquids of the economy enjoys this property.

2d. That the pancreatic juice in emulsionizing and in modifying the fatty matters renders them absorbable and in this way becomes the indispensable and only agent of the formation of chyle.—*Gazette Médicale.*—D.W.Y.

